# Supplementary material for: Real-time concrete strength monitoring using piezoelectric sensors and deep learning
Source: Nat Commun. 2025 Dec 12;17:473. doi: 10.1038/s41467-025-67168-8 (PMC12800108; doi:10.1038/s41467-025-67168-8)
Supplement: Supplementary file 1 — Supplementary Information [file 41467_2025_67168_MOESM1_ESM.pdf]

Supplementary Information

Real-Time Concrete Strength Monitoring Using Piezoelectric Sensors and Deep Learning

Guangshuai Han,[a] Yen-Fang Su,[a,b] Rui He,[a] Cihang Huang ,[a] Zhihao Kong ,[a] Guang Lin,[c,d] Yining Feng,[a]* and Na Lu[a]*

[a] Lyles School of Civil and Construction Engineering, Purdue University, West Lafayette, IN, 47907, USA.

[b] Department of Civil and Environmental Engineering, Louisiana State University, Baton Rouge, LA 70803, USA.

[c] Department of Mathematics, Purdue University, West Lafayette, IN, 47907, USA.

[d] School of Mechanical Engineering, Purdue University, West Lafayette, IN 47907, USA.

*Corresponding author: Yining Feng(feng109@purdue.edu); Na Lu (luna@purdue.edu).

- SUPPLMENTARY FIGURES（S1–S24）
- SUPPLMENTARY TABLES（S1–S4）
- SUPPLMENTARY METHODS
- REFERENCES

SUPPLEMENTARY FIGURES

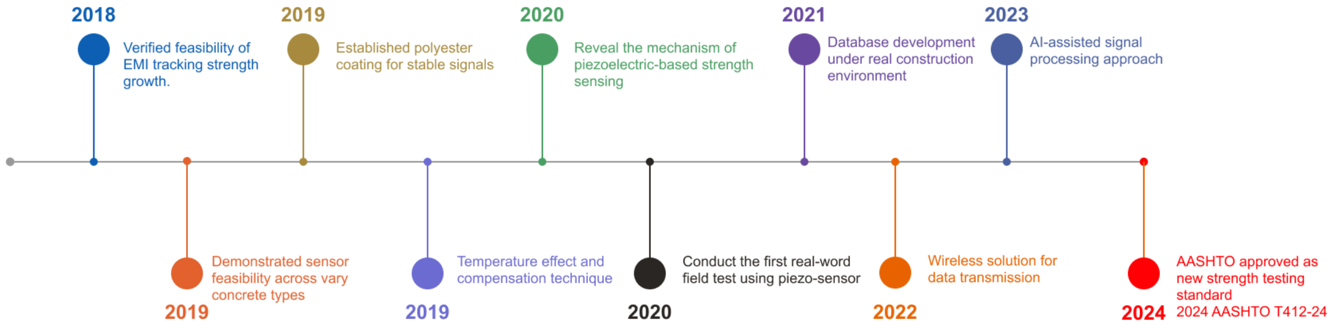

Fig. S1 Roadmap of concrete strength sensing development over a 7-year period. The timeline illustrates the transition from early laboratory feasibility studies to full-scale sensor deployment in real-world construction projects.

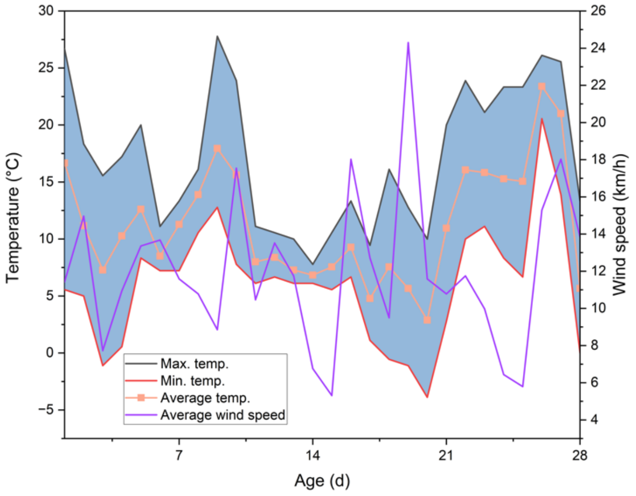

slab 1&2

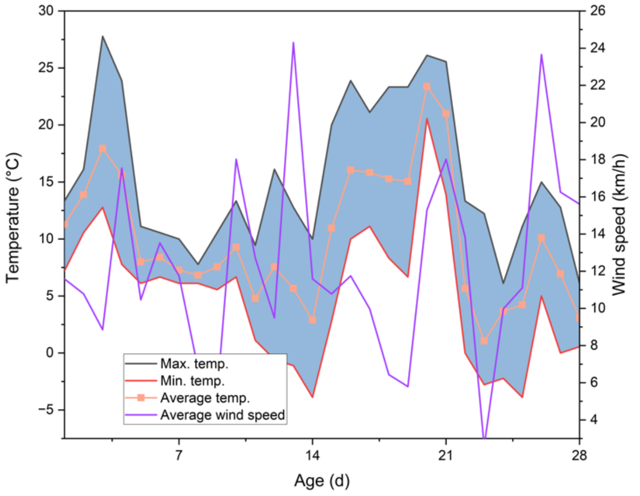

slab 3&4

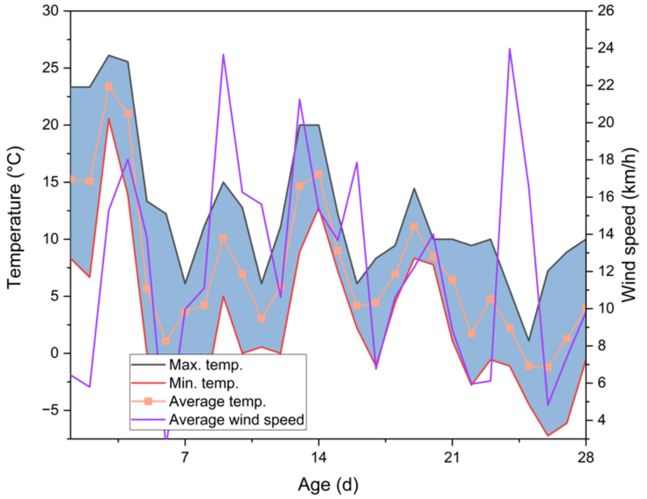

Slab 5, 6, &7

Fig. S2 Environmental conditions during concrete slab casting, including ambient temperature and wind speed, recorded for each of the three casting dates. These factors contribute to variations in the curing process and are considered in the analysis of strength development and EMI signal behavior.

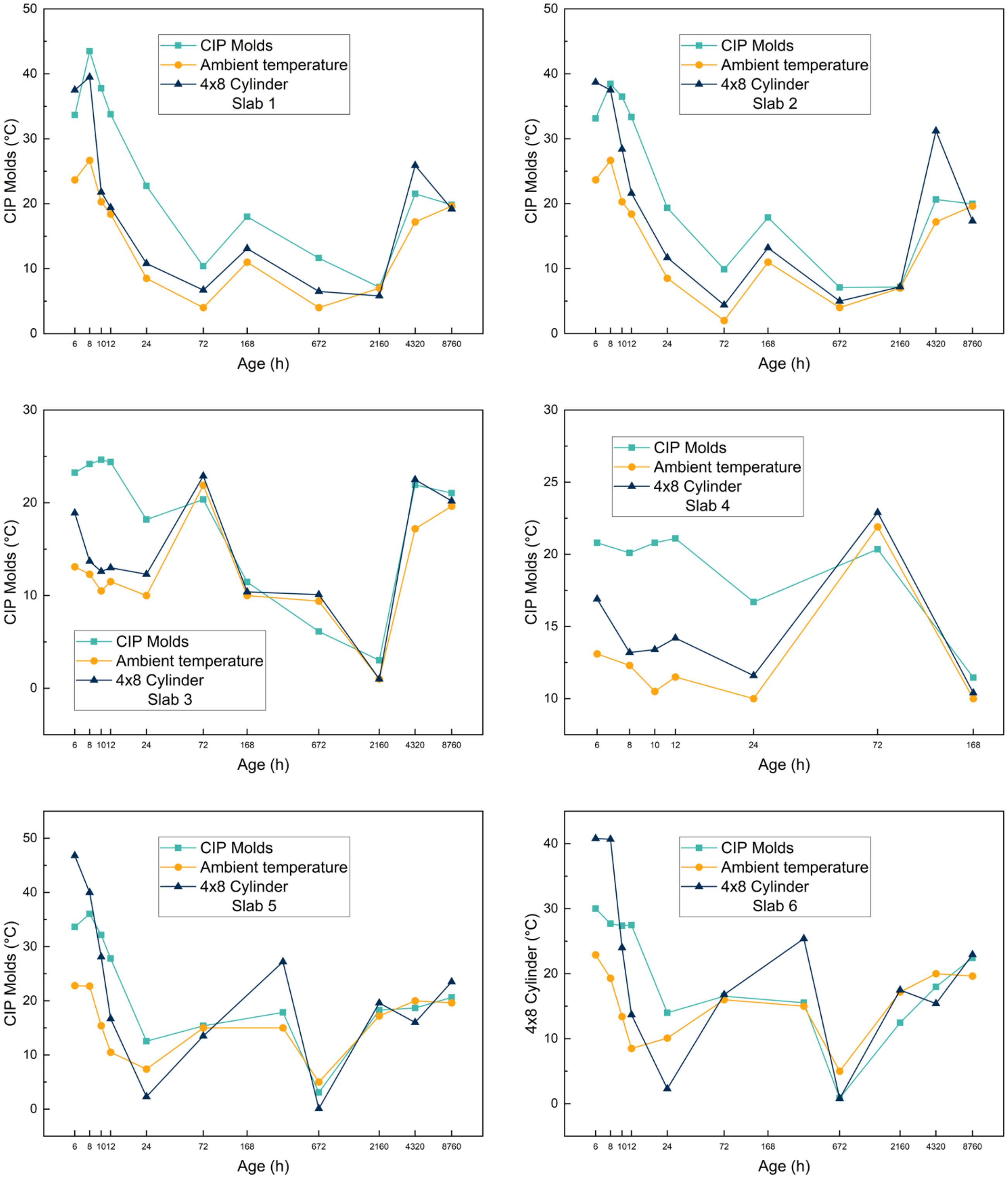
CIP Molds
Ambient temperature
4x8 Cylinder
Slab 1
Slab 2
Slab 3
Slab 4
Slab 5
Slab 6
CIP Molds (°C)
4x8 Cylinder (°C)
Age (h)
6
8
10
12
24
72
168
672
2160
4320
8760

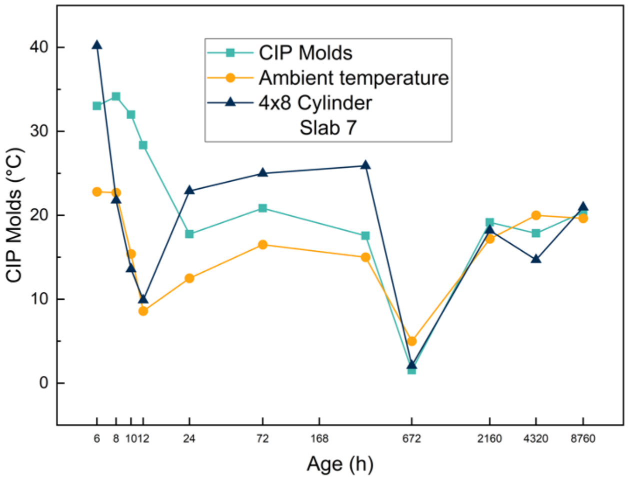

Fig. S3 Temperature profiles of cast-in-place (CIP) molds and 4x8 cylinder samples compared with ambient temperature over a one-year period.

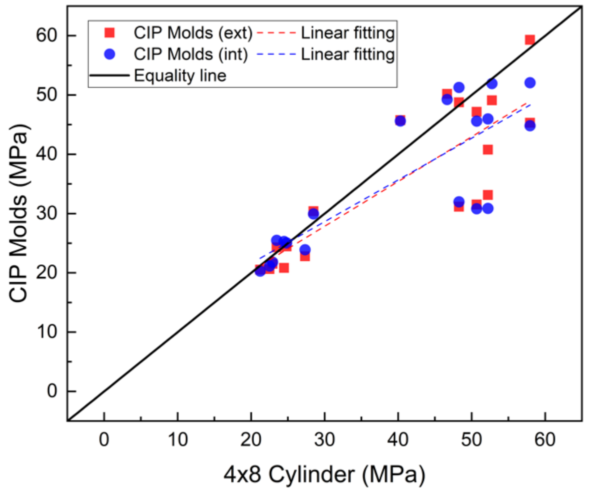

(a)

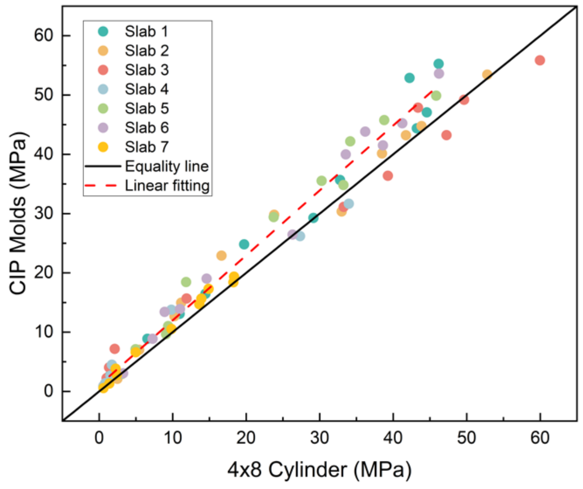

(b)

Fig. S4 Comparison of compressive strength between CIP molds and 4x8 cylinder samples. (a) reported by Alabama DOT; (b) samples from this study samples across seven different slabs.

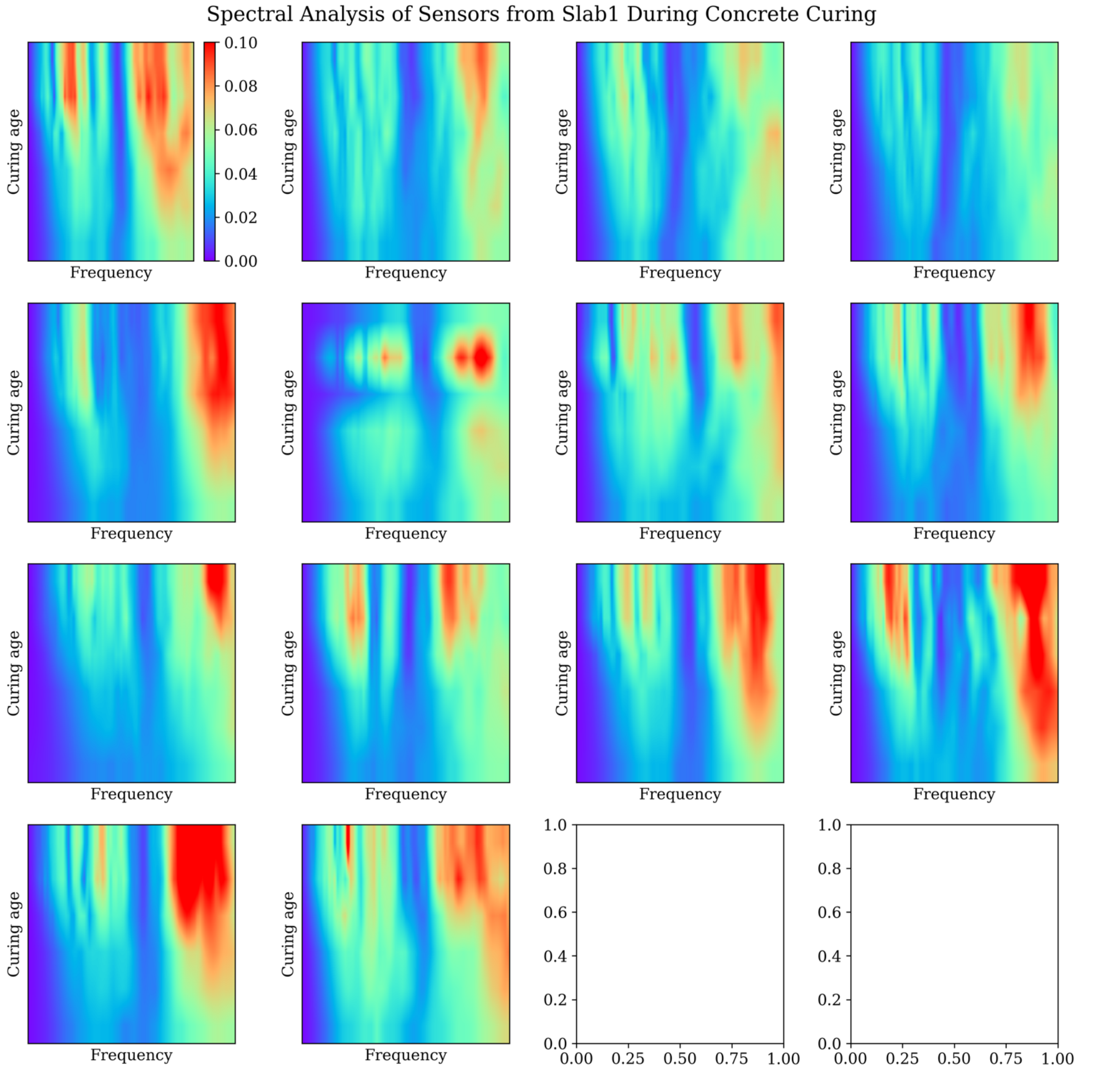

Fig. S5 Real part of the EMI signal (conductance) for Slab 1. Each figure illustrates the evolution of the real part of the piezoelectric sensor's EMI signal over time, capturing changes in frequency response as the concrete slabs cured during the one-year observation period.

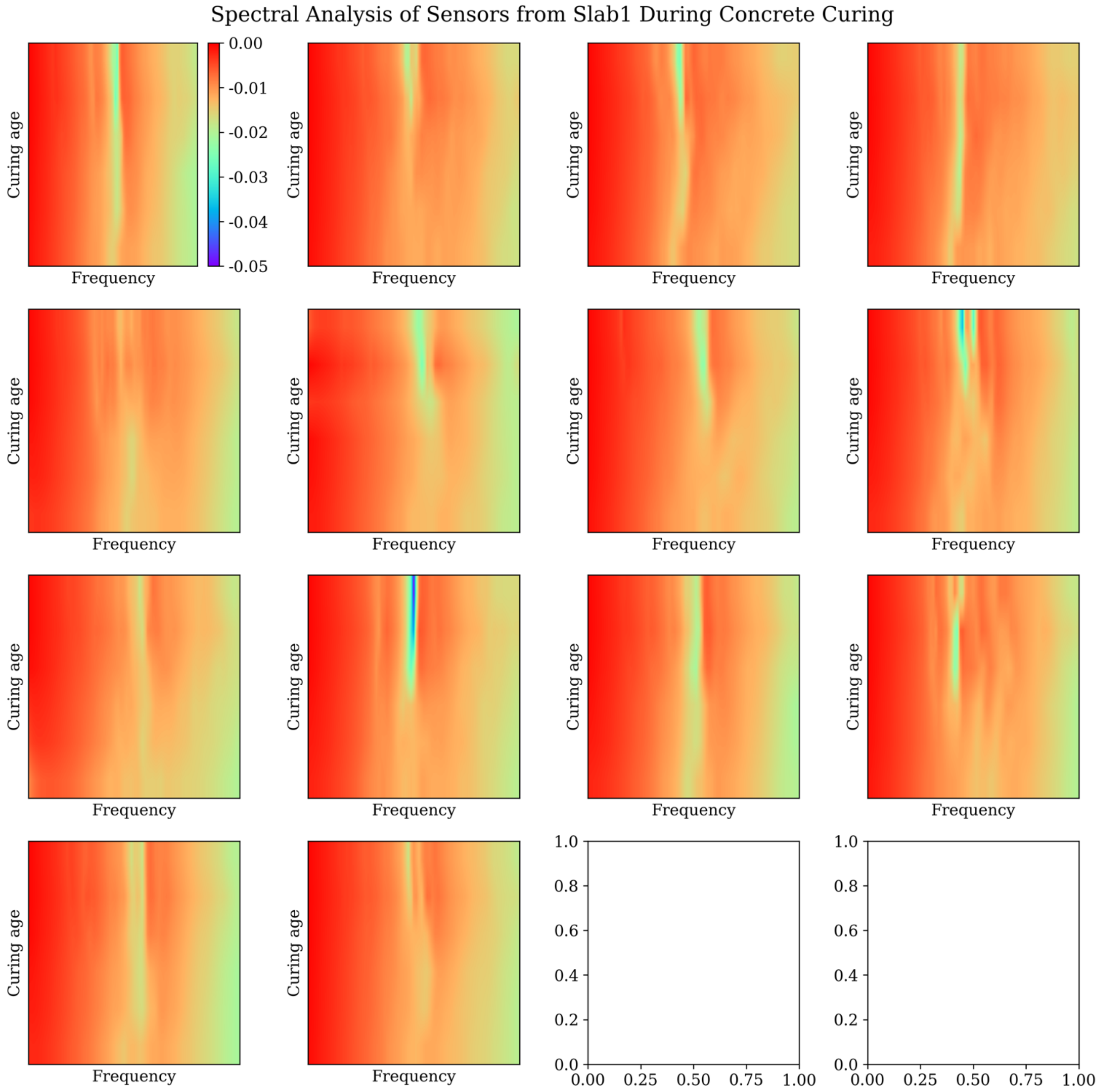

Fig. S6 Imaginary part of the EMI signal (susceptance) for Slab 1. Each figure illustrates the evolution of the imaginary part of the piezoelectric sensor's EMI signal over time, capturing changes in frequency response as the concrete slabs cured during the one-year observation period.

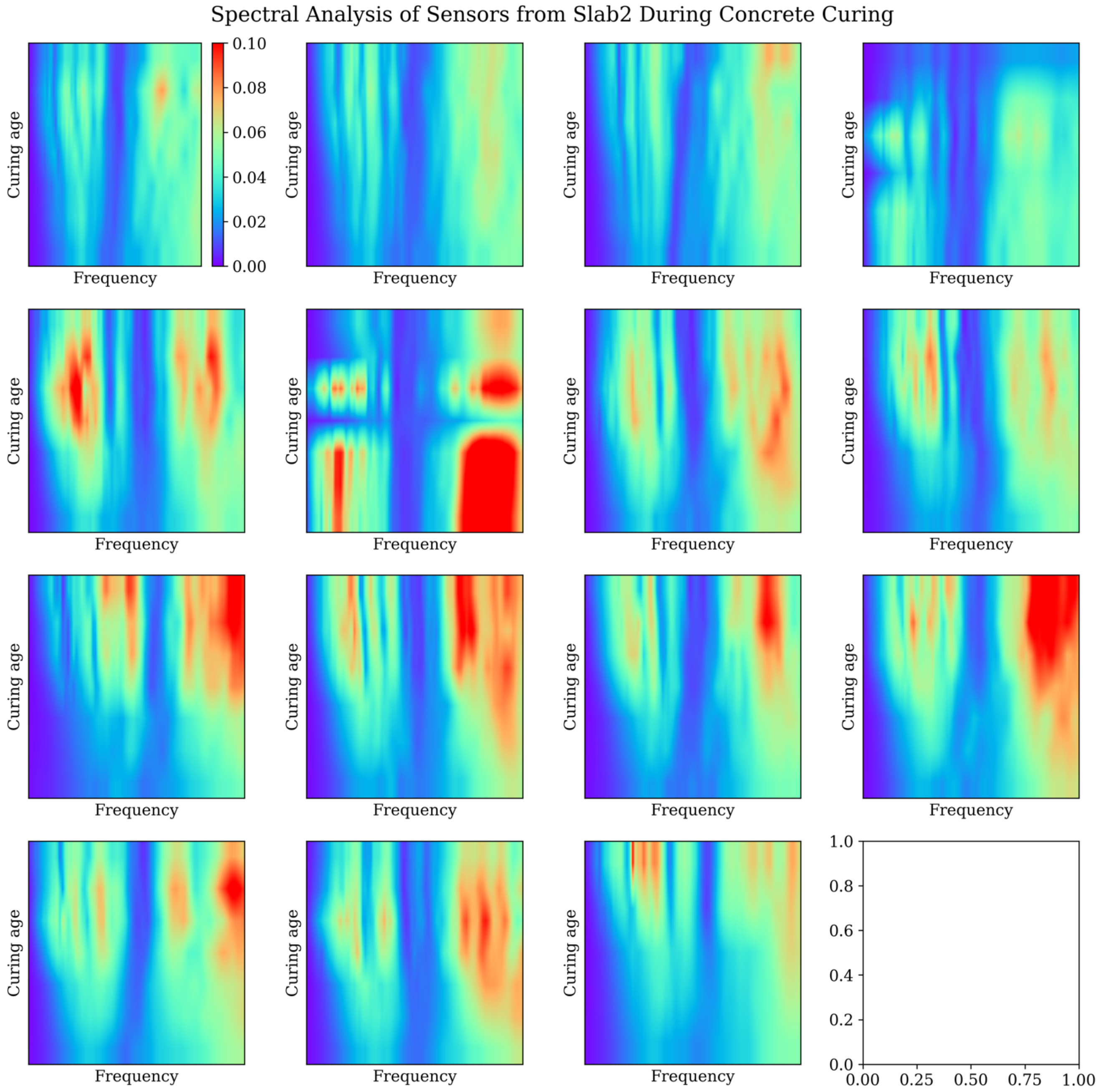

Fig. S7 Real part of the EMI signal (conductance) for Slab 2. Each figure illustrates the evolution of the real part of the piezoelectric sensor's EMI signal over time, capturing changes in frequency response as the concrete slabs cured during the one-year observation period.

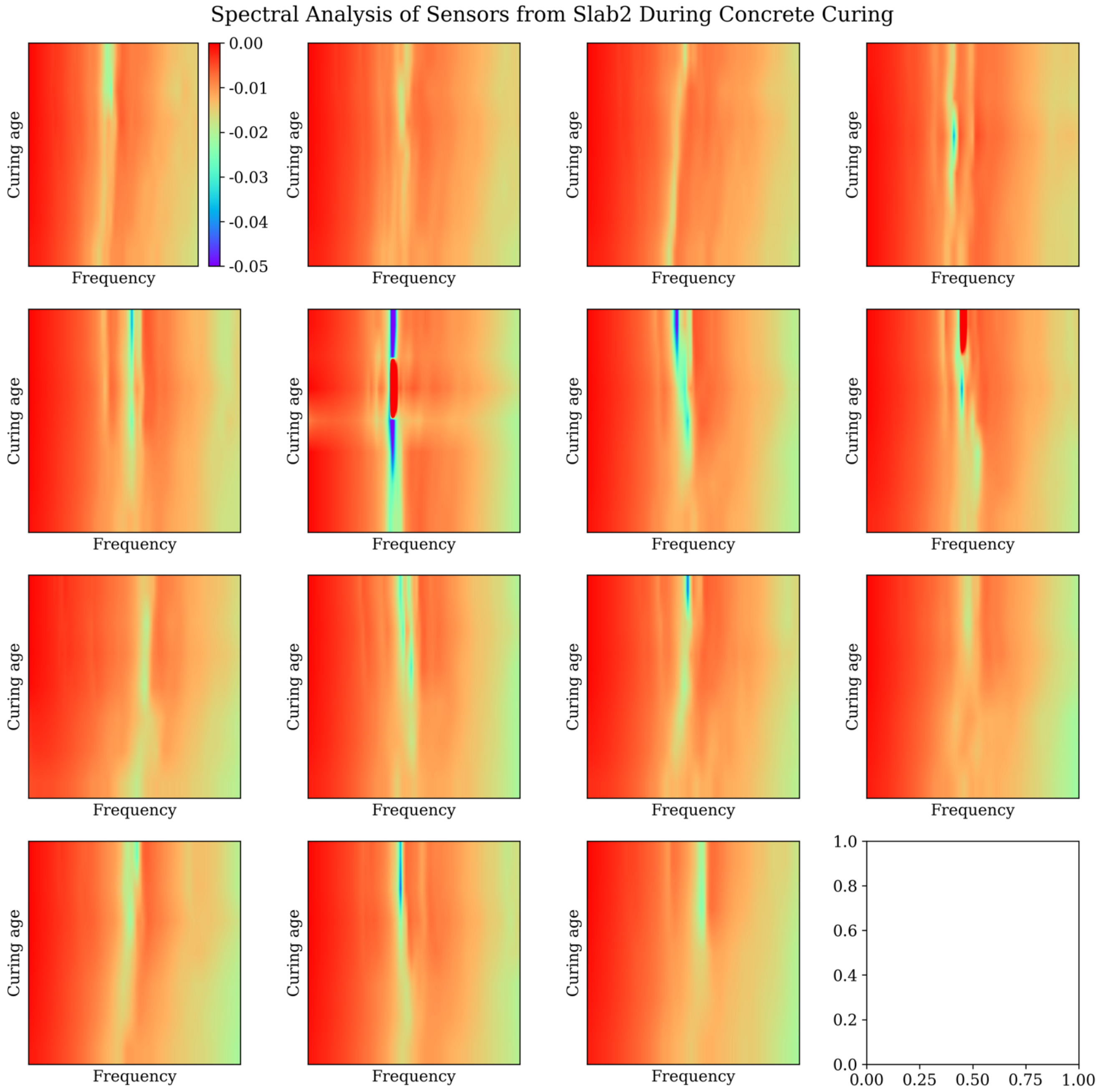

Fig. S8 Imaginary part of the EMI signal (susceptance) for Slab 2. Each figure illustrates the evolution of the imaginary part of the piezoelectric sensor's EMI signal over time, capturing changes in frequency response as the concrete slabs cured during the one-year observation period.

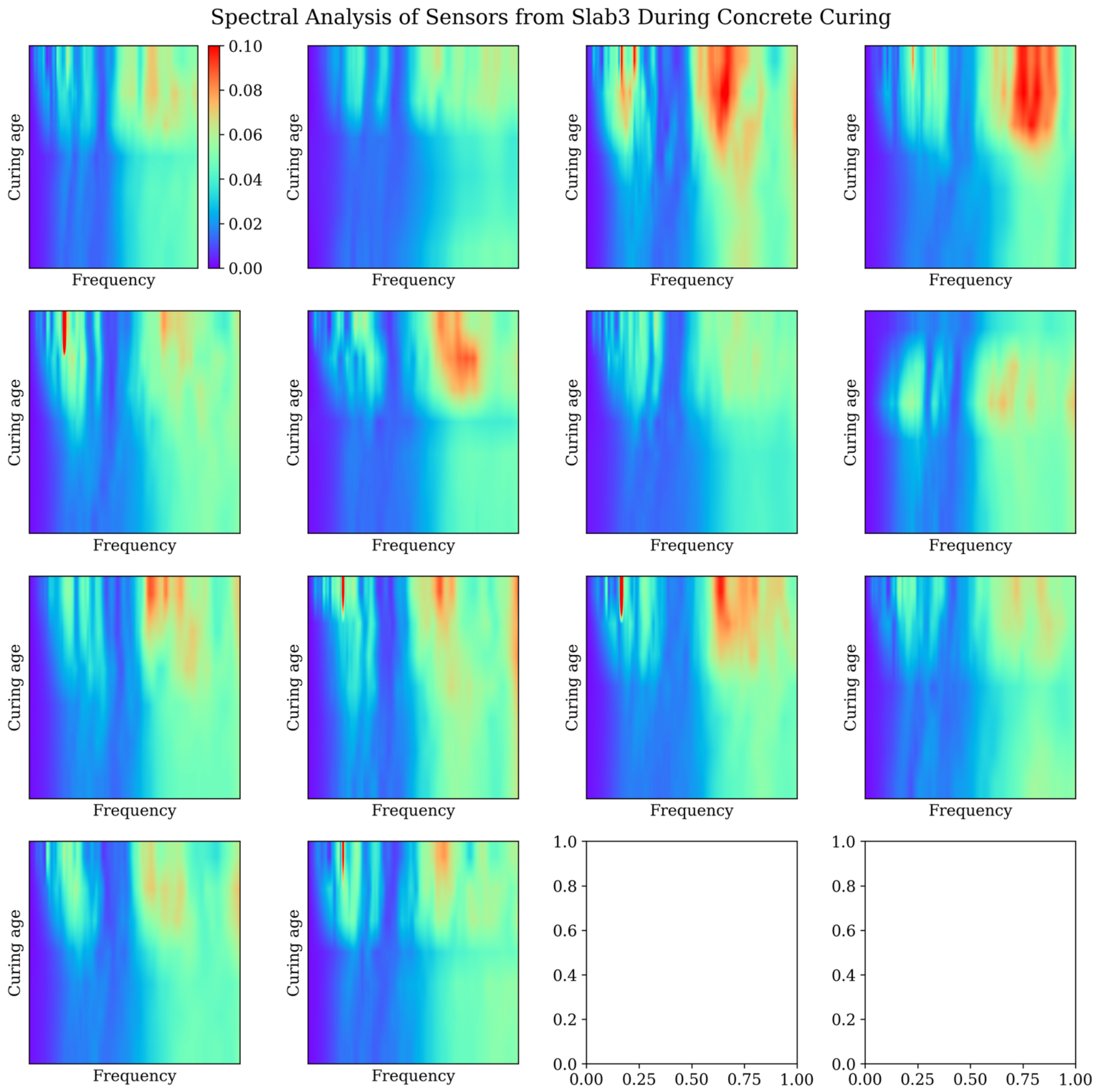

Fig. S9 Real part of the EMI signal (conductance) for Slab 3. Each figure illustrates the evolution of the real part of the piezoelectric sensor's EMI signal over time, capturing changes in frequency response as the concrete slabs cured during the one-year observation period.

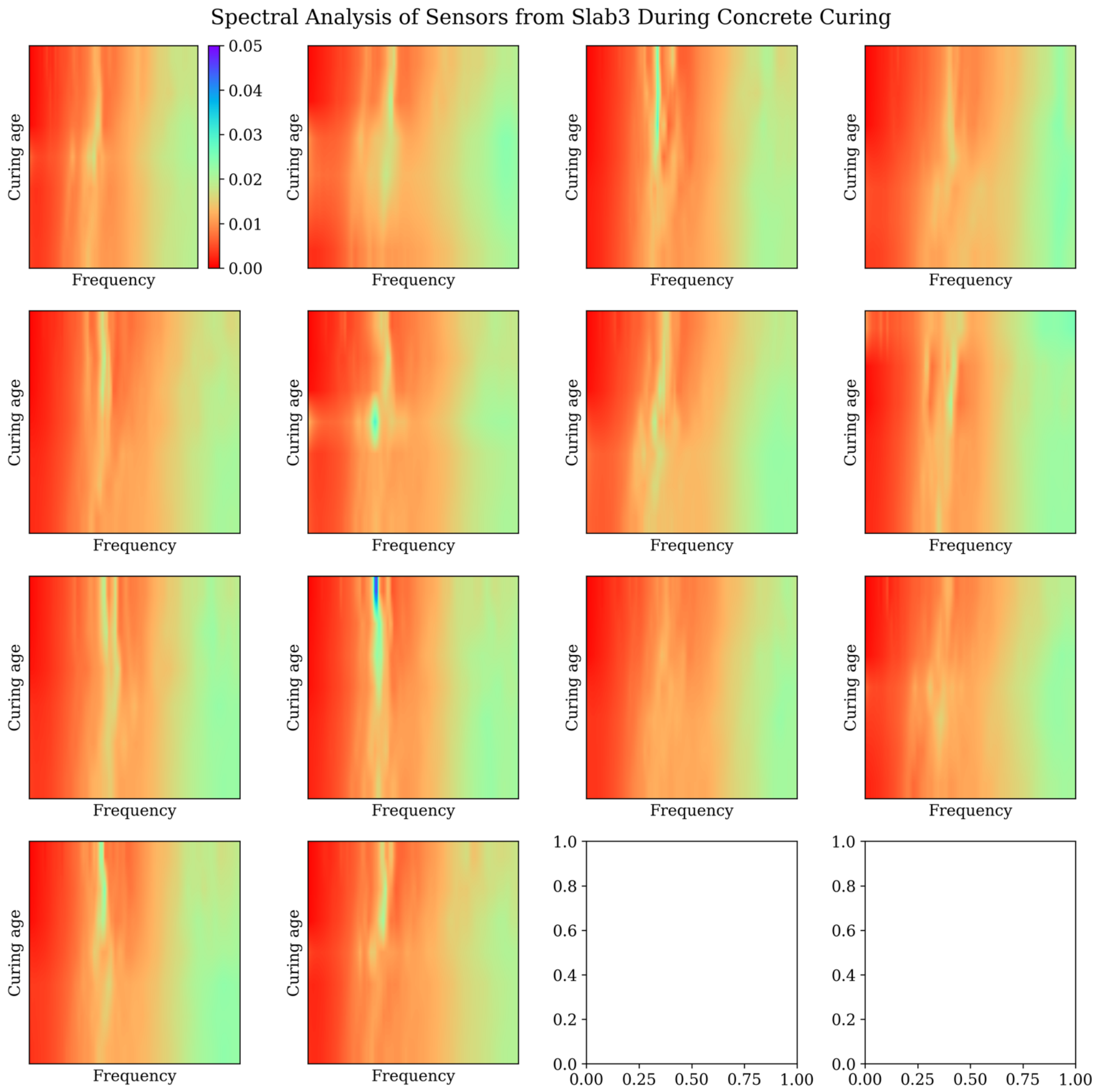

Fig. S10 Imaginary part of the EMI signal (susceptance) for Slab 3. Each figure illustrates the evolution of the imaginary part of the piezoelectric sensor's EMI signal over time, capturing changes in frequency response as the concrete slabs cured during the one-year observation period.

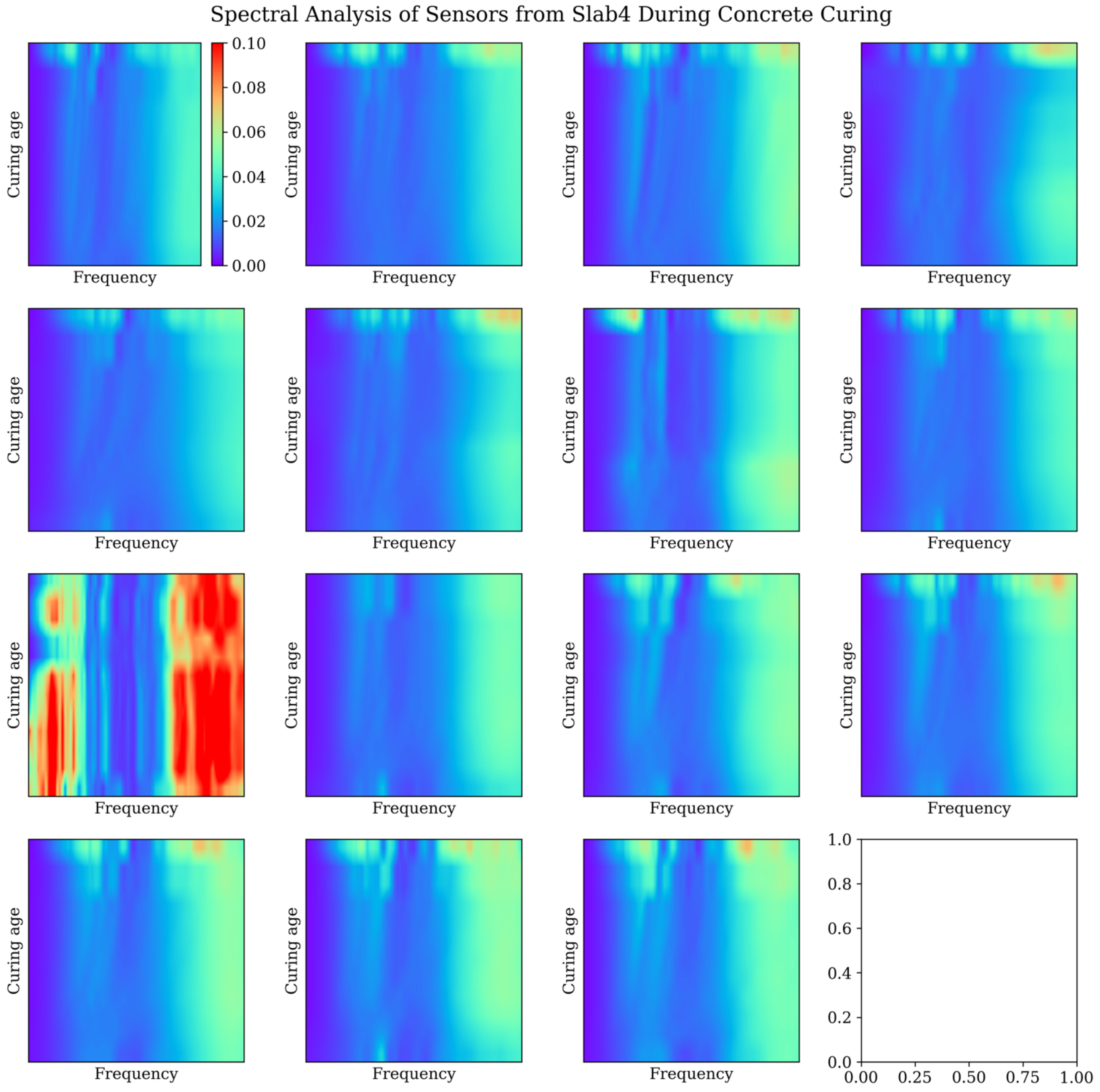

Fig. S11 Real part of the EMI signal (conductance) for Slab 4. Each figure illustrates the evolution of the real part of the piezoelectric sensor's EMI signal over time, capturing changes in frequency response as the concrete slabs cured during the one-year observation period.

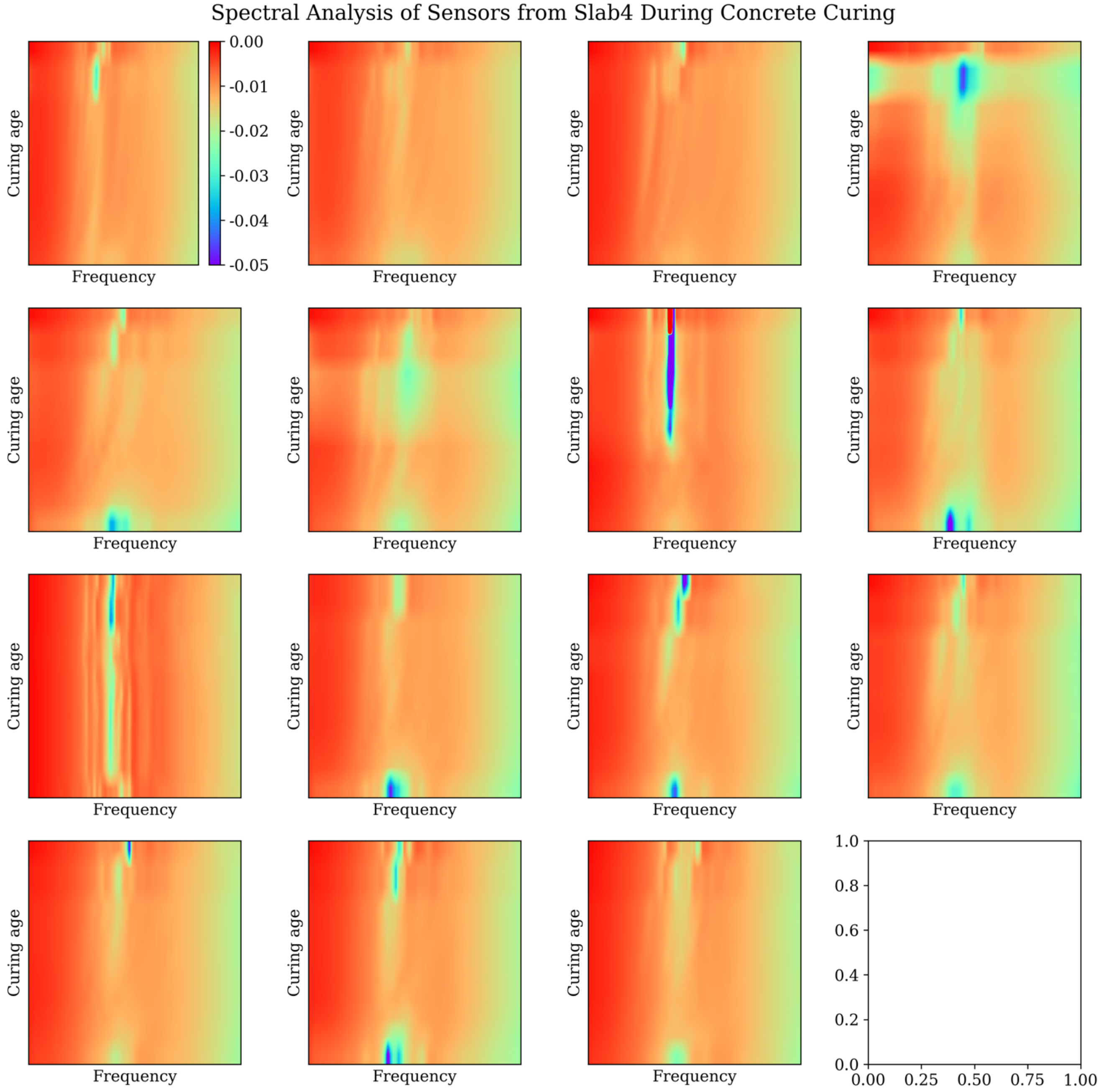

Fig. S12 Imaginary part of the EMI signal (susceptance) for Slab 4. Each figure illustrates the evolution of the imaginary part of the piezoelectric sensor's EMI signal over time, capturing changes in frequency response as the concrete slabs cured during the one-year observation period.

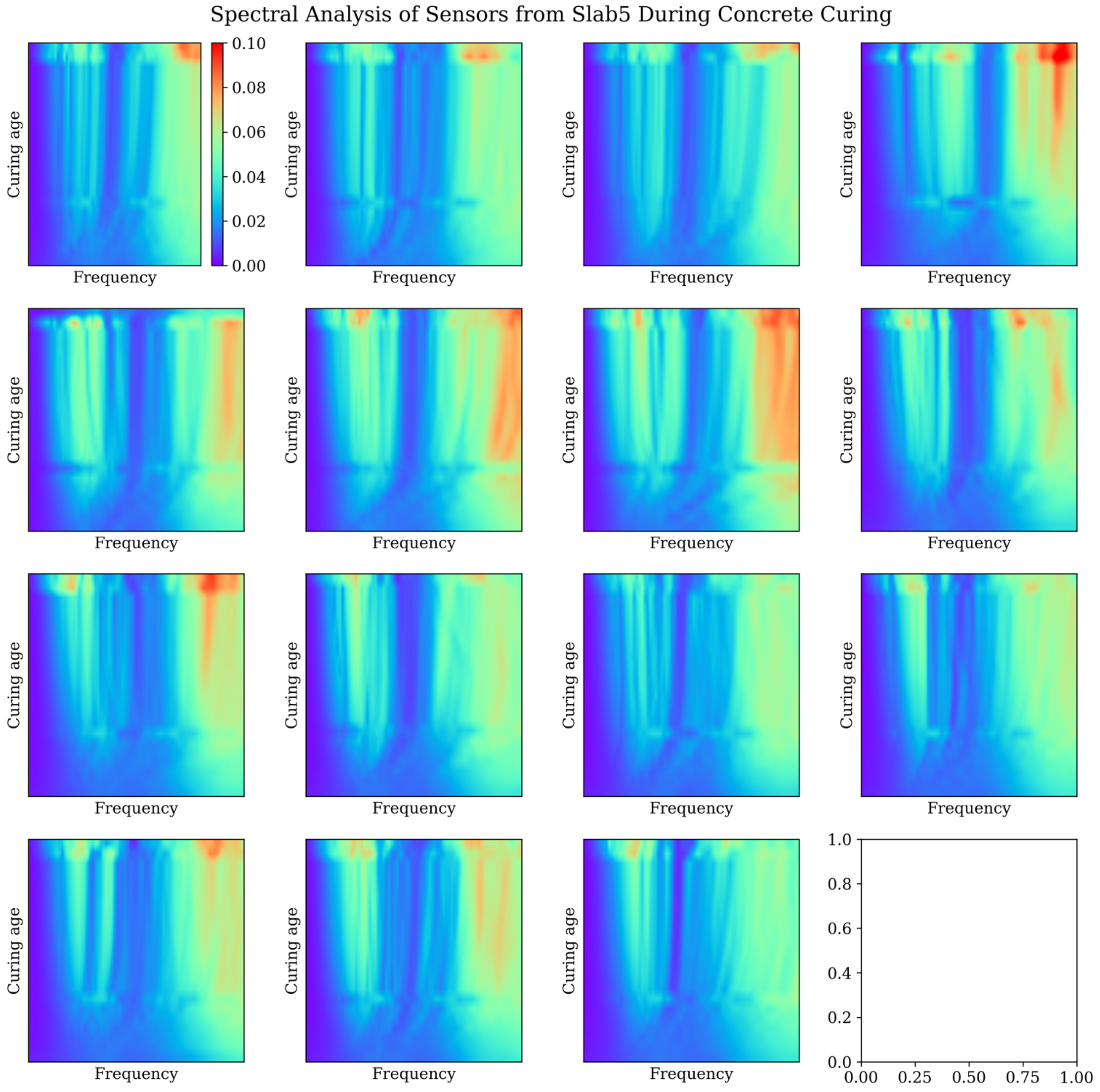

Fig. S13 Real part of the EMI signal (conductance) for Slab 5. Each figure illustrates the evolution of the real part of the piezoelectric sensor's EMI signal over time, capturing changes in frequency response as the concrete slabs cured during the one-year observation period.

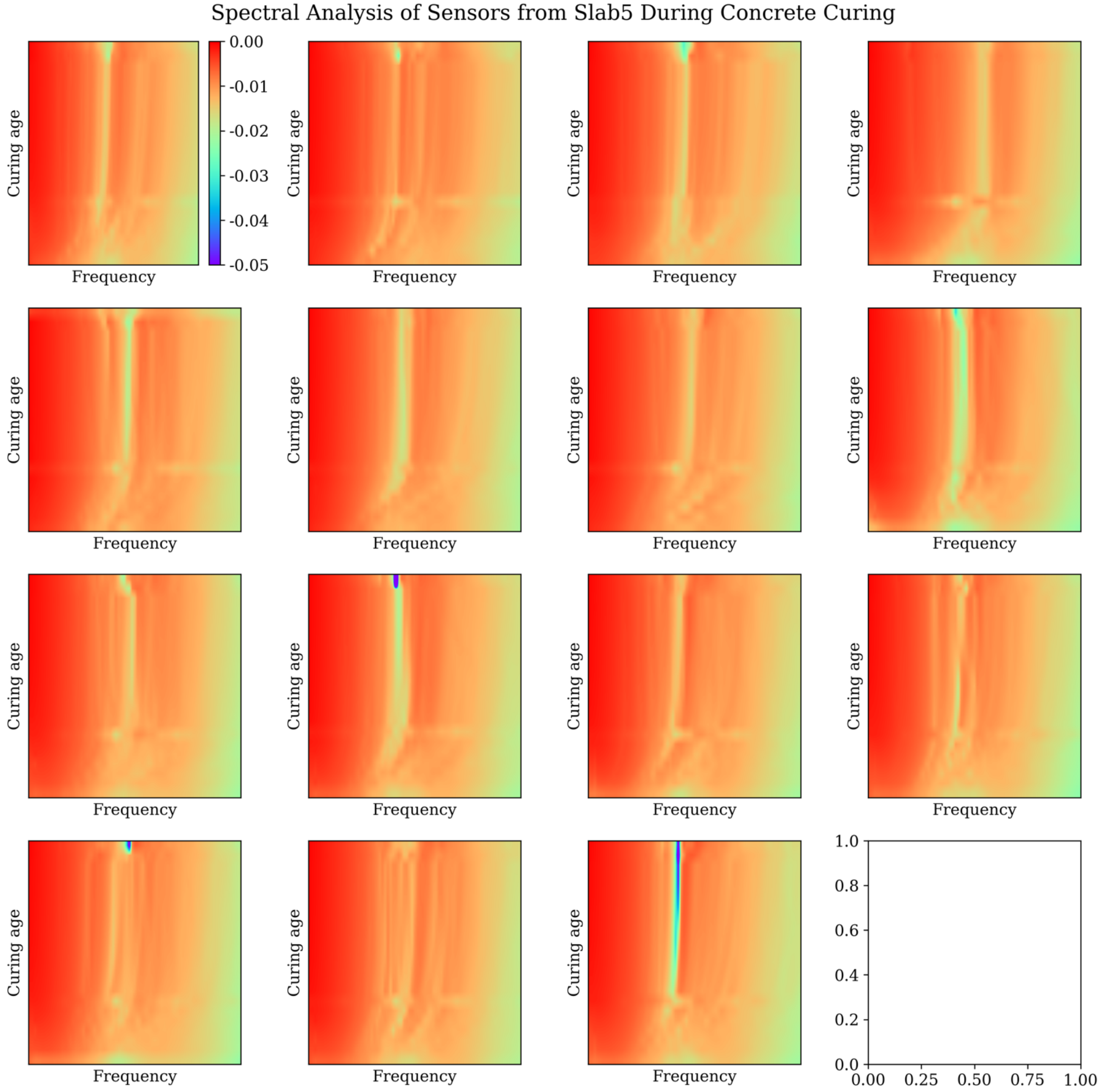

Fig. S14 Imaginary part of the EMI signal (susceptance) for Slab 5. Each figure illustrates the evolution of the imaginary part of the piezoelectric sensor's EMI signal over time, capturing changes in frequency response as the concrete slabs cured during the one-year observation period.

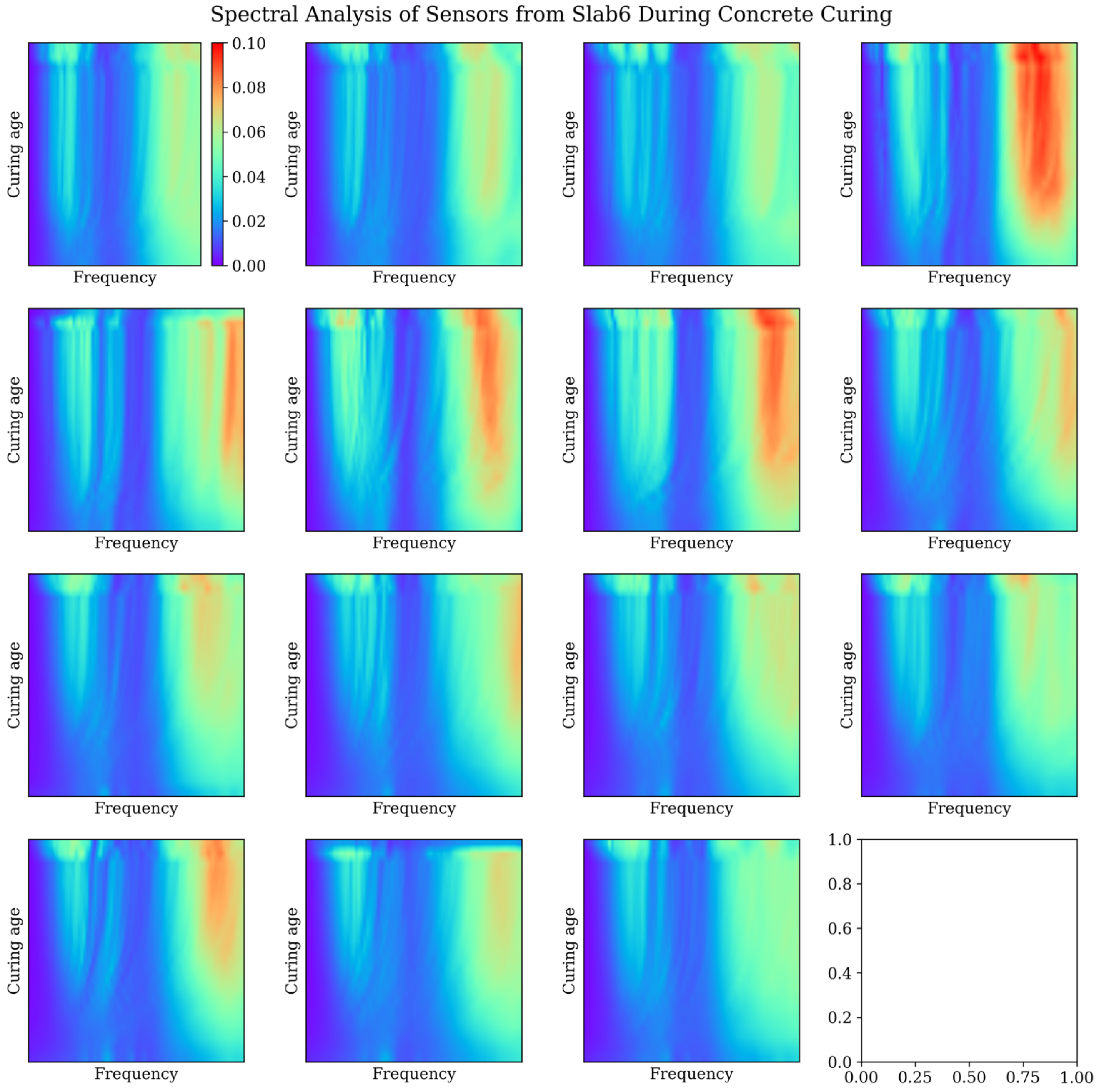

Fig. S15 Real part of the EMI signal (conductance) for Slab 6. Each figure illustrates the evolution of the real part of the piezoelectric sensor's EMI signal over time, capturing changes in frequency response as the concrete slabs cured during the one-year observation period.

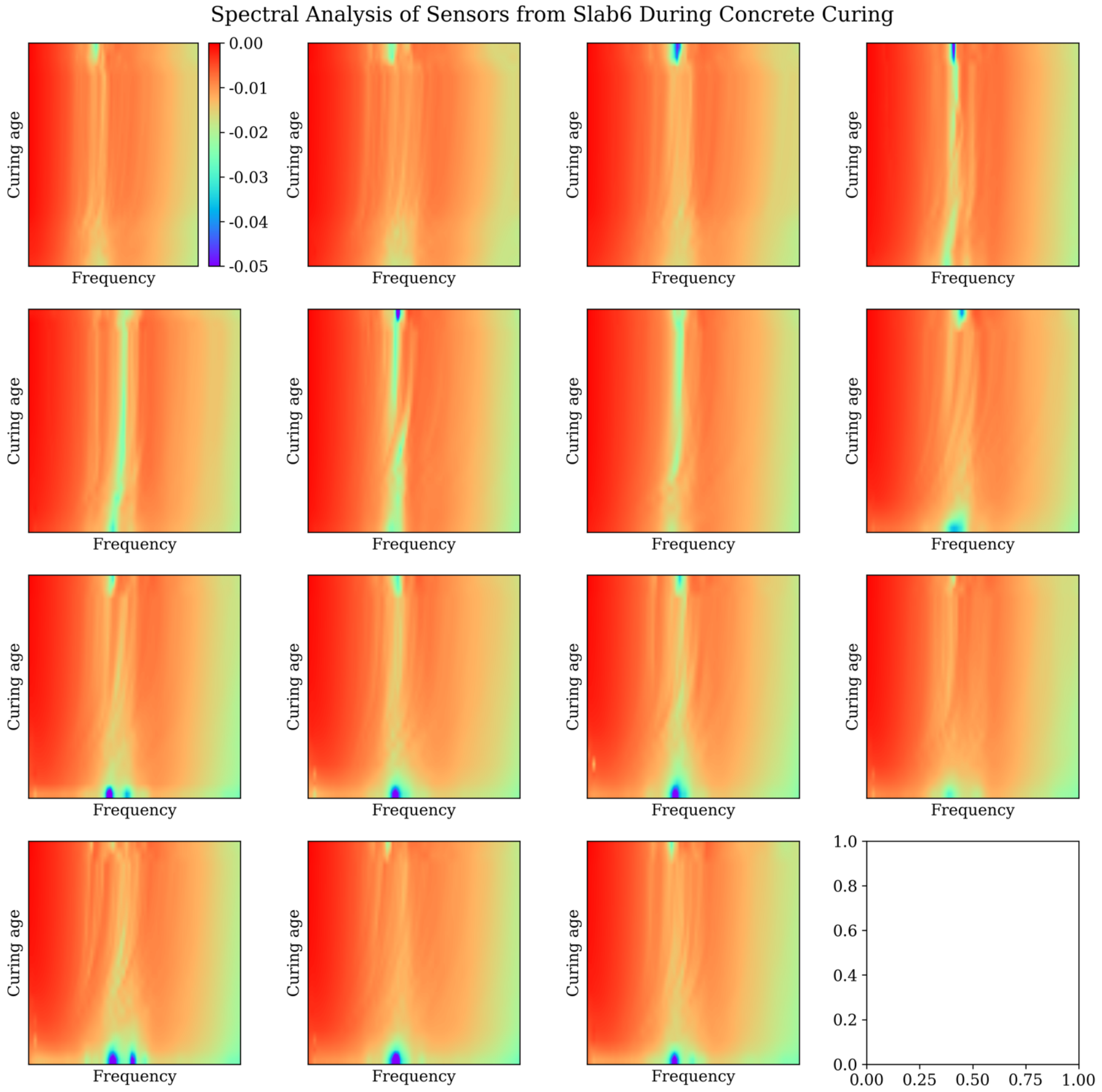

Fig. S16 Imaginary part of the EMI signal (susceptance) for Slab 6. Each figure illustrates the evolution of the imaginary part of the piezoelectric sensor's EMI signal over time, capturing changes in frequency response as the concrete slabs cured during the one-year observation period.

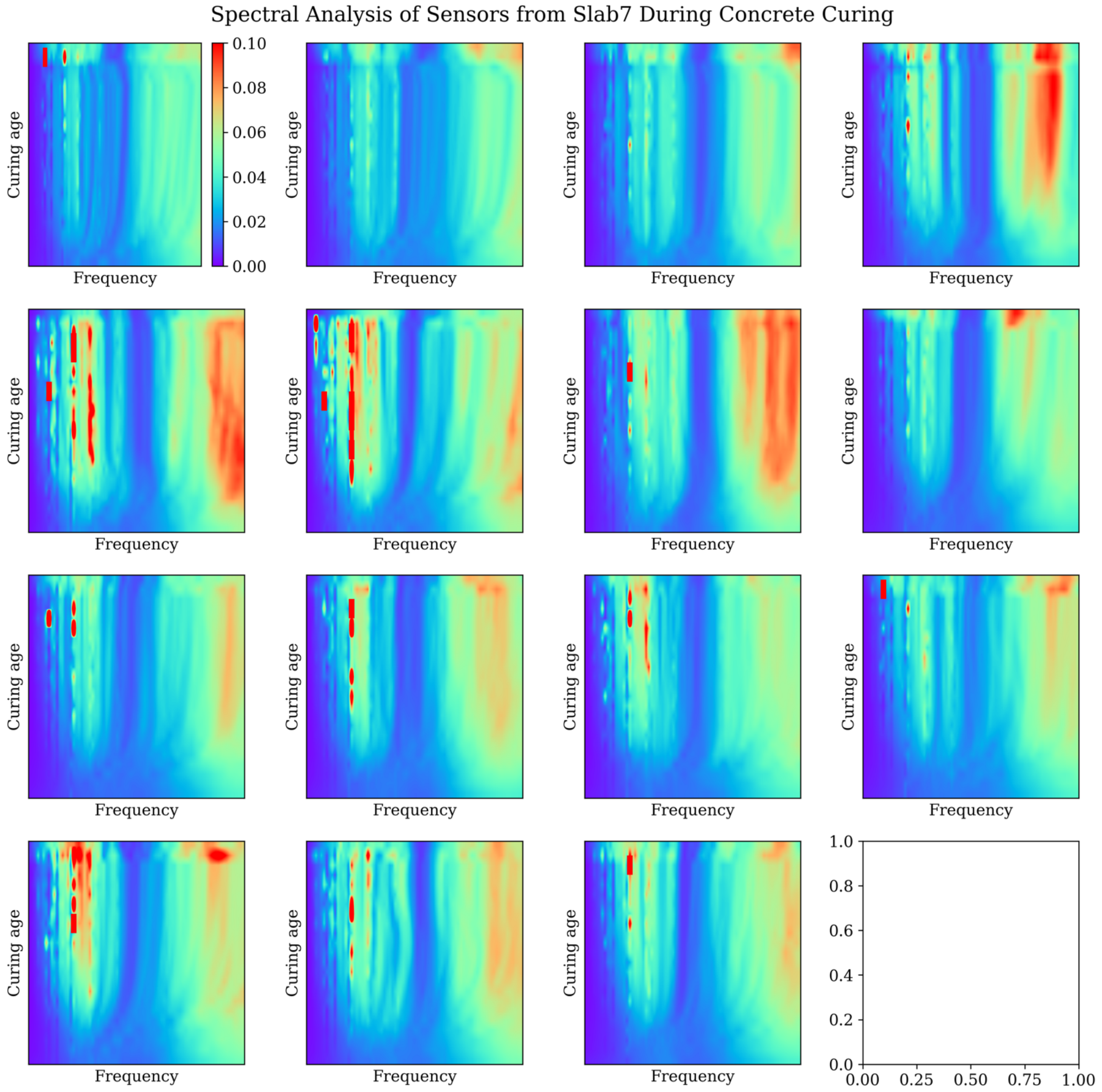

Fig. S17 Real part of the EMI signal (conductance) for Slab 7. Each figure illustrates the evolution of the real part of the piezoelectric sensor's EMI signal over time, capturing changes in frequency response as the concrete slabs cured during the one-year observation period.

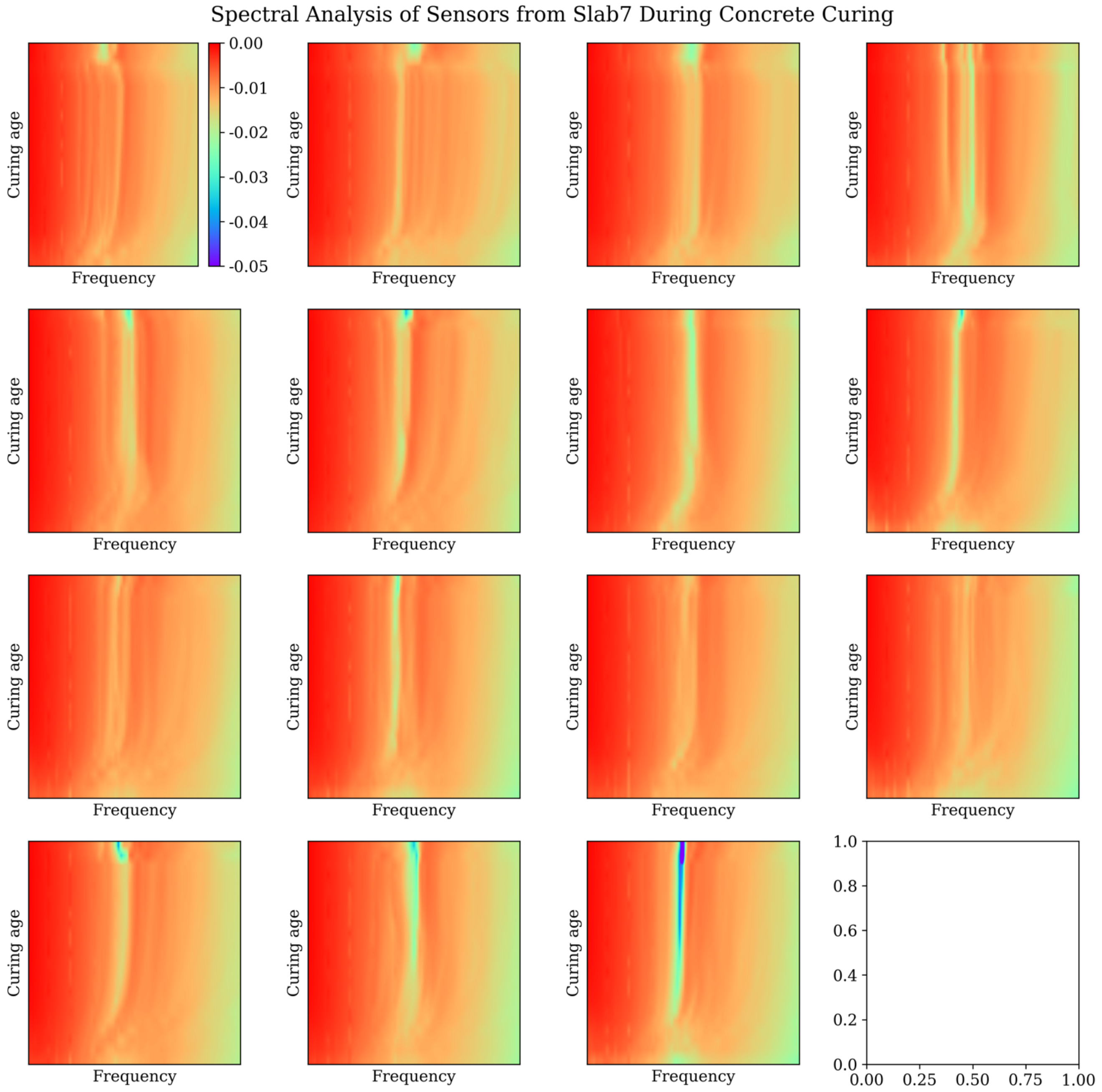

Fig. S18 Imaginary part of the EMI signal (susceptance) for Slab 7. Each figure illustrates the evolution of the imaginary part of the piezoelectric sensor's EMI signal over time, capturing changes in frequency response as the concrete slabs cured during the one-year observation period.

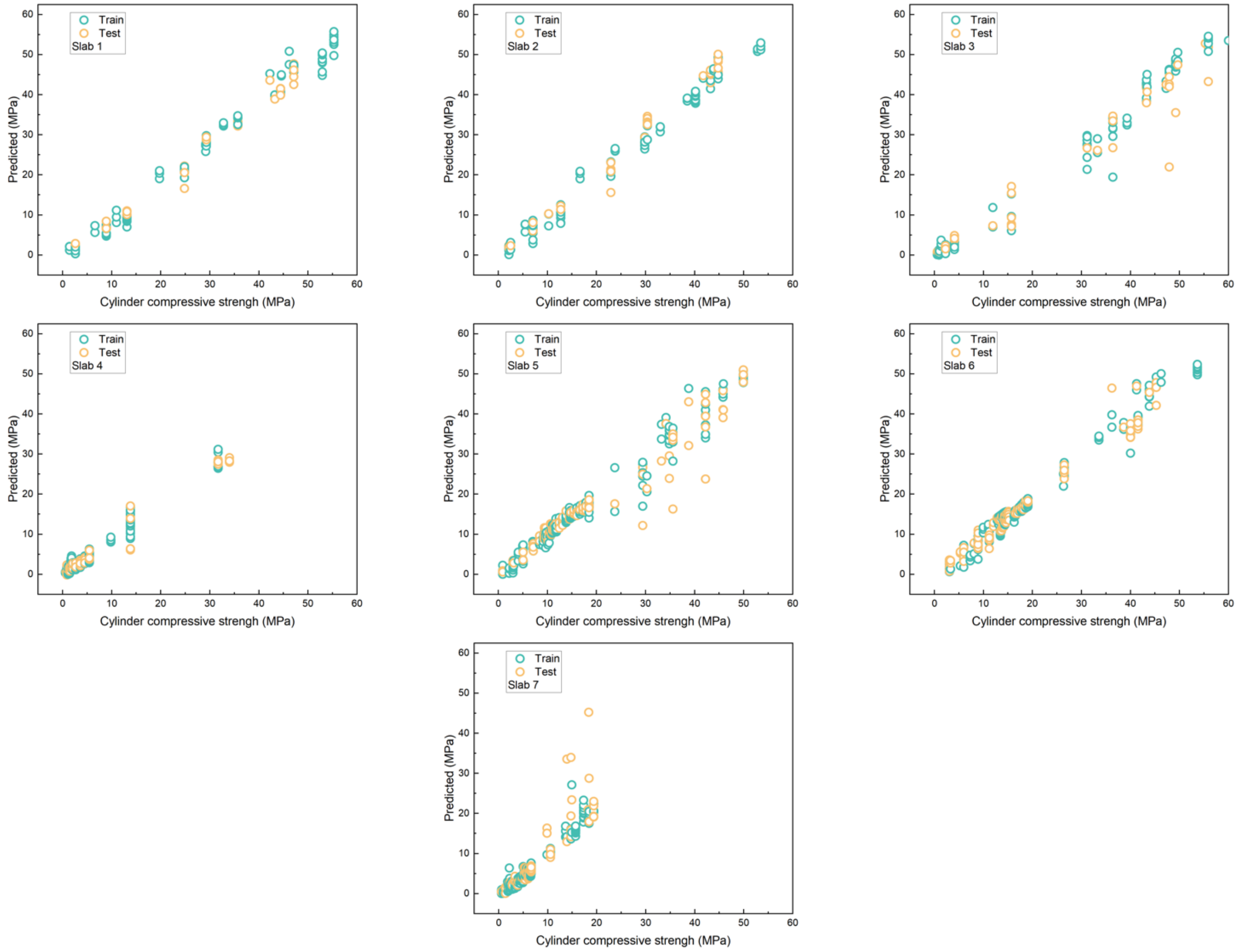

Fig. S19 Performance of the AI model in predicting concrete strength for each slab. The figure shows the strength prediction results for each of the seven concrete slabs using the proposed baseline mechanism and 1D CNN model. The model demonstrates consistent accuracy across different concrete types, highlighting its reliability and robustness in predicting the strength of various concrete structures.

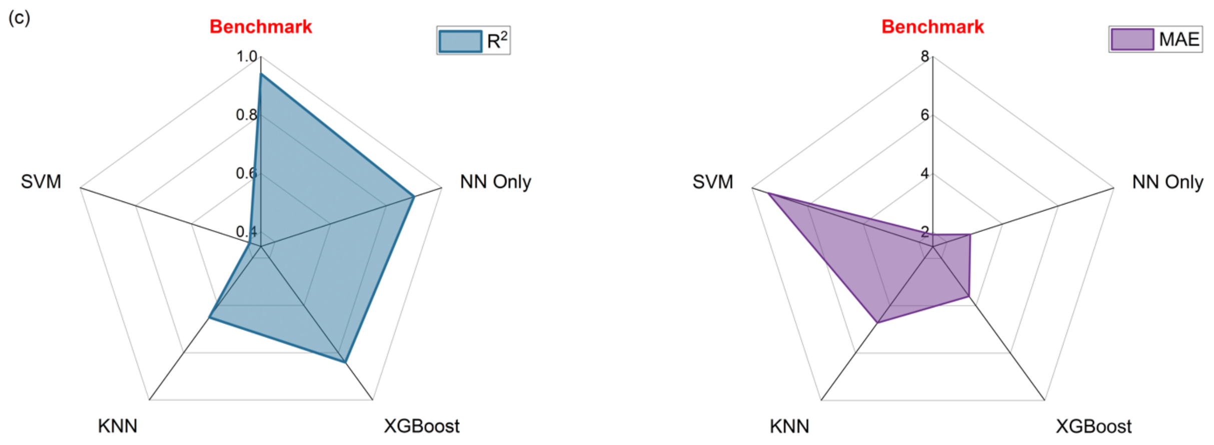

Fig. S20 Comparison of different machine learning algorithms for EMI signal processing. (a) R^2 comparison; outperforms the others. (b) MAE comparison.

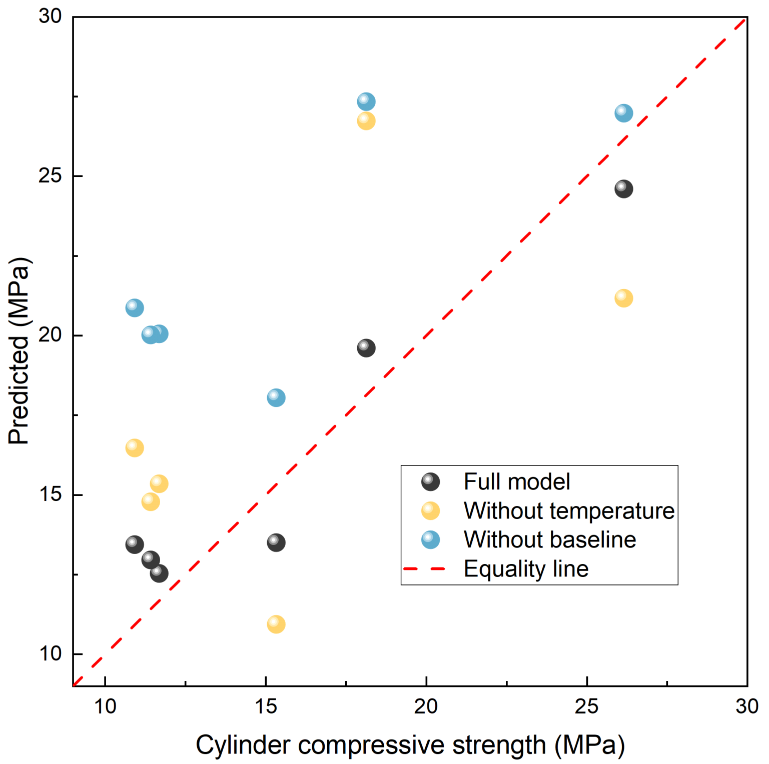

Fig. S21 Comparison of model performance without baseline and without temperature input on different field testing data. Both models deviate significantly from the benchmark, with increased prediction errors, underscoring the importance of baseline and temperature features in achieving accurate strength predictions.

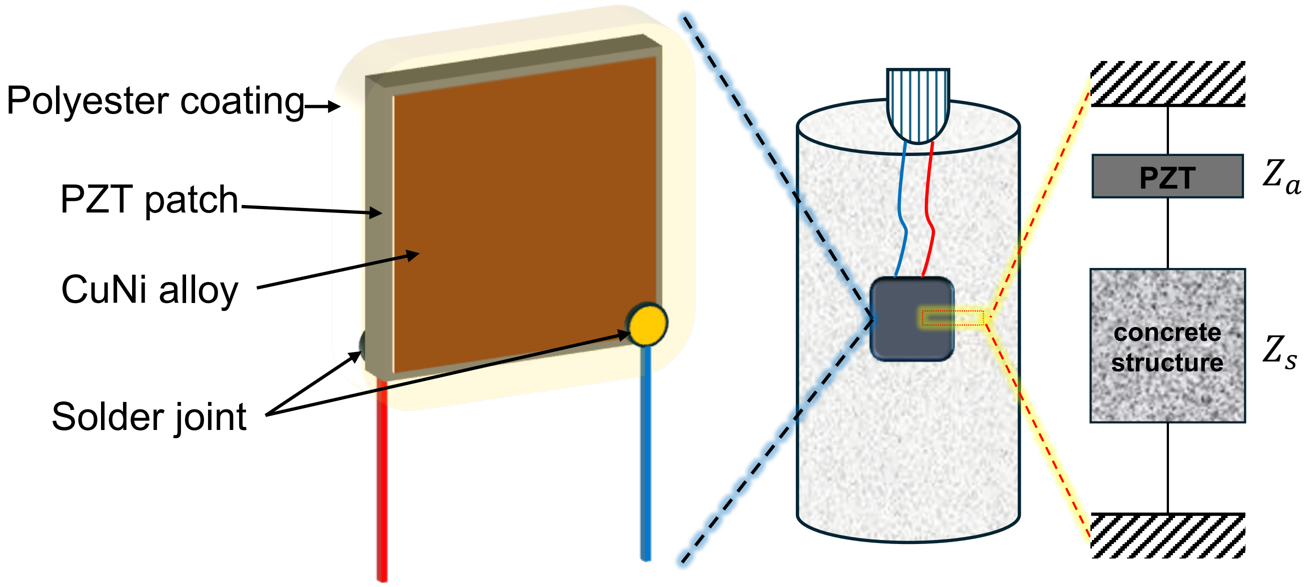

Fig. S22 PZT sensor fabrication, installation, and its coupling with concrete structure.

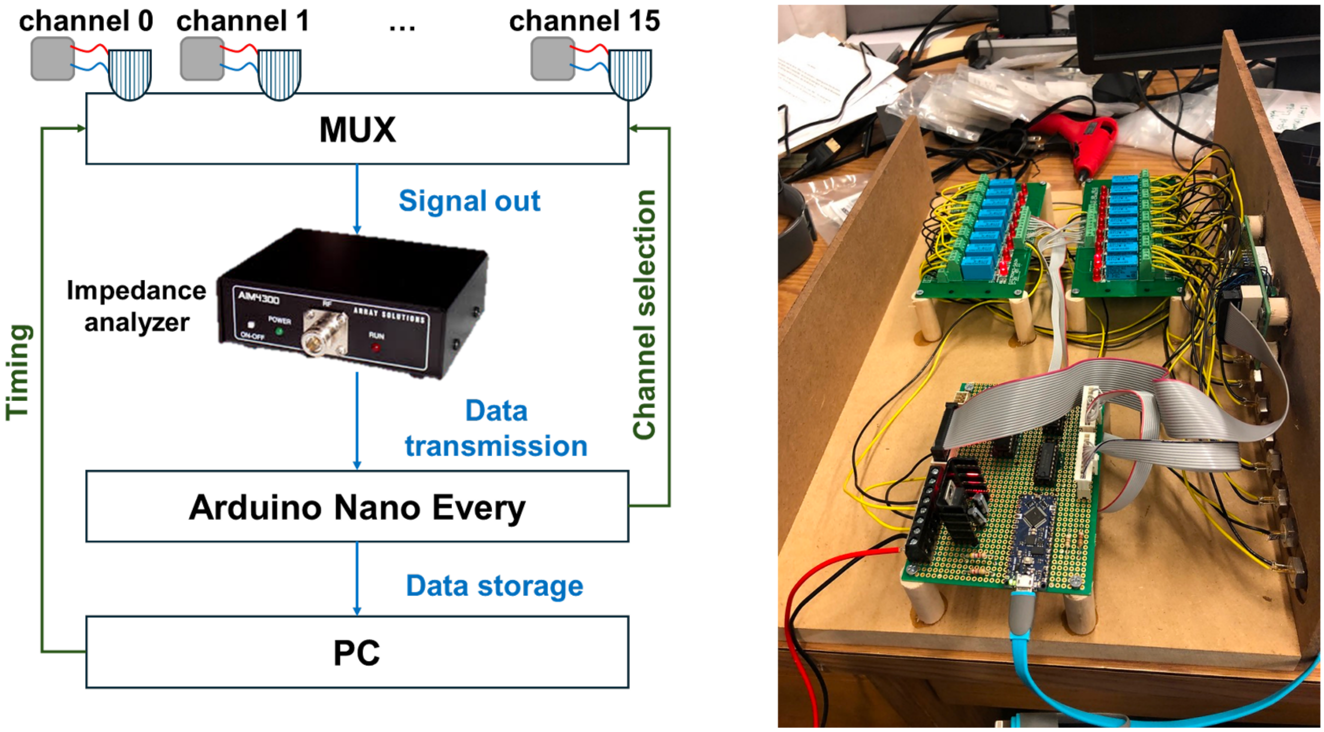

Fig. S23 Schematic of the multi-channel signal collection signal

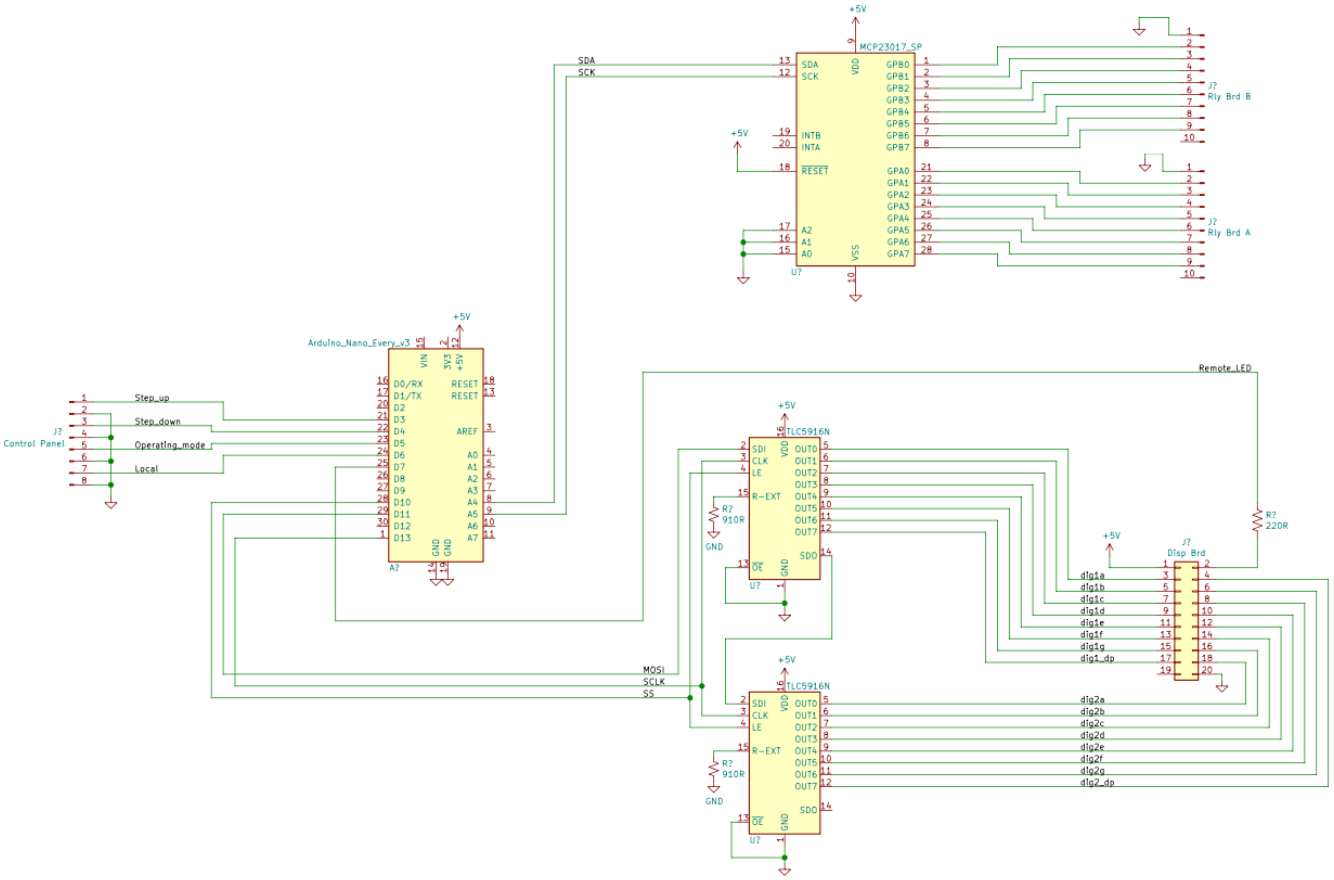

Fig. S24 Circuit design of the MUX

SUPPLEMENTARY TABLES

Table S1 Detailed mix design(lbs/cubic yards) parameters for each of the seven concrete slabs, including water-to-cement (W/C) ratios, types and quantities of admixtures, and supplementary cementitious materials (SCMs). Each slab features a distinct combination of materials to capture a wide range of strength development behaviors. All designs were based on a target 28-day compressive strength of 4,000 psi (approximately 27.58 MPa).

Slab No.	Cement	#8 Gravel AP	Natural Sand	Water reducer(oz)	Water(gal)	Nano-silica(oz)	Fly ash	28 days compressive strength (MPa)
1	563	1927	1506	38.6	16.6	22.6	0	43.21
2	563	1927	1506	38.6	18.6	22.6	0	38.48
3	564	1893	1513	22.6	21.2	22.6	0	47.26
4	450	1927	1506	22.6	18.6	0	113	\
5	568	1920	1506	11.3	16.8	0	0	33.23
6	582	1880	1493	22.3	18.3	0	0	38.60
7	558	1893	1493	22.6	22.0	0	0	13.90

Table S2 Best model parameters and performance for different input feature combinations. The table shows the optimal parameters identified through grid search for different input feature combinations. The output channels are listed in sequence, representing the first convolutional layer, second convolutional layer, first fully connected layer, and second fully connected layer.

Frequency range (kHz)	Baseline	Temperature input	Learning rate	Optimizer	Batch size	Output channels	R^2	MAE(MPa)
10-200	√	√	0.0005	Adam	16	64, 128, 64, 32	0.94	1.91
10-500	√	√	0.0005	Adam	16	32, 128, 64, 32	0.90	2.69
200-500	√	√	0.001	Adam	32	64, 64, 64, 32	0.85	3.47
10-200		√	0.001	Adam	16	64, 128, 64, 32	0.84	3.58
10-500		√	0.0005	Adam	16	32,128, 64, 32	0.81	3.95
200-500		√	0.0001	SGD	16	32, 128, 64, 32	0.75	4.17
10-200	√		0.001	Adam	16	32, 64, 64, 32	0.90	2.40
10-500	√		0.0005	Adam	16	64, 128, 64, 32	0.85	3.43
200-500	√		0.001	Adam	32	32, 128, 64, 32	0.82	3.91

Table S3 Summary of field test results: sensor usage and prediction accuracy.

Task	No. of sensor usage	Mean absolute error (MPa)	Mean percentage error (%)
I74@06/12/2019	6	2.44	13.8
I74@08/03/2019	10	1.13	7.13
I465@07/20/2019	7	1.54	13.5
I465@09/10/2019	8	2.52	23.01

Table S4 Field test summary showing curing age, temperature, cylinder testing results, AI model predictions, and sensor deviation.

Task	Curing age	Temperature	Cylinder testing	Average	Standard deviation
I74@06/12/2019	5.00	38.37		2.57	0.61
	6.00	39.33		4.32	0.15
	7.00	36.30		5.54	0.25
	8.00	35.60		6.66	0.19
	24.00	24.43	15.33	13.50	0.36
	72.00	23.73	26.15	24.60	1.26
I74@08/03/2019	5.00	32.70		2.25	0.06
	6.00	34.25		3.88	0.10
	7.00	37.35		5.43	0.12
	8.00	38.90		6.22	0.06
	24.00	36.55	11.68	12.54	0.24
	72.00	26.55	18.13	19.60	1.73
I465@07/20/2019	5.00	56.13		4.44	0.03
	6.00	56.43		4.90	0.04
	7.00	48.90		5.04	0.05
	8.00	48.70		5.63	0.06
	24.00	43.00	11.42	12.96	0.17
I465@09/10/2019	5.00	51.73		4.10	0.07
	6.00	54.93		4.92	0.08
	7.00	53.35		5.35	0.08
	8.00	53.10		5.86	0.10
	24.00	43.07	10.92	13.44	0.33

SUPPLEMENTARY METHODS

S1. Sensor Design and Working Mechanism

The piezoelectric sensor used in this study is based on a PZT ceramic plate (PQYY+0412), fabricated from lead zirconate titanate (PZT) with dimensions of 10 mm × 10 mm in width and length, and 0.2 mm in thickness. The electrodes are formed by sputter-coating both sides of the PZT plate with a CuNi (Copper-Nickel) alloy, serving as the core signal transmission elements. To ensure durable electrical connection, cold soldering was performed using silver epoxy, which minimizes thermal damage to the piezoelectric material. As shown in Fig. S22, the sensing unit is then encapsulated using a polyester coating, which offers mechanical protection and insulation when embedded in concrete. This design has been validated in previous studies[1] to preserve the sensor's electromechanical sensitivity while withstanding the harsh alkaline and high-humidity environment during concrete curing.

The integrated sensing mechanism relies on the EMI (Electro-Mechanical Impedance) principle, in which the PZT transducer serves as both an actuator and a sensor. When excited by a swept sinusoidal voltage signal, the sensor generates localized vibrations that interact with the surrounding concrete. The response is then recorded as an EMI signature, which reflects the mechanical impedance of the host structure. Changes in concrete stiffness and mass properties—associated with strength development—alter the EMI spectrum, thus enabling non-destructive monitoring of structural evolution in real time. This electromechanical interaction can be simplified into a one-dimensional model, as illustrated on the right side of Fig. S22. The underlying principle is that when the PZT sensor is bonded to a host structure, its electrical admittance response is influenced by the mechanical impedance of the surrounding material through electromechanical coupling. As concrete undergoes hydration, its mechanical impedance evolves due to changes in stiffness and mass. This variation is transferred to the PZT sensor and is reflected as a shift or amplitude change in the EMI spectrum. Therefore, by continuously monitoring the EMI signal during early-age curing, we can effectively capture the strength development of the concrete structure.

S2. Signal Acquisition System Configuration

To support high-throughput EMI measurements across multiple embedded sensors, we developed a custom-built multiplexer (MUX) system interfaced with the AIM 4300 Impedance Analyzer (Array Solutions). As shown in the schematic and hardware photos (Fig. S23 and Fig. S24), the MUX supports up to 16 sensor input channels and enables sequential excitation and acquisition of signals from each sensor. The switching control is managed by an Arduino Nano Every (ABX00033), programmed to

autonomously coordinate timing, signal routing, and data synchronization. The relays, actuated by logic commands, are governed via an I²C-controlled GPIO expander (MCP23017) and cascaded shift registers (74HC595), allowing efficient addressable selection of each input channel. A Python-based host-side controller handles USB serial communication and timing, modeled after legacy ASCII-based protocols to ensure reliability and flexibility in field operations. To minimize signal degradation and environmental interference, the entire electronics system—including relays, Arduino controller, and supporting power circuitry—was housed in a shielded ValuLine metal chassis. This design mitigated electrical noise and provided robust protection from temperature variation, dust, and moisture during outdoor deployment.

Each measurement cycle involved applying an alternating current (AC) excitation signal of 1 V peak-to-peak to the bonded PZT patch. The impedance spectrum was scanned across a frequency range of 10 kHz to 500 kHz, with a resolution of 5 kHz, enabling high-fidelity capture of frequency-dependent EMI response. The multiplexer maintained a crosstalk level below 0.04%, and although mechanical relay switching introduced a latency of ~10–20 ms, this had negligible impact on the resolution and timing of the EMI measurements.

S3. Statistical Index for EMI Signal Analysis

To establish a baseline understanding of the EMI signal variation during strength development, we employed the Root Mean Square Deviation (RMSD) index, which quantifies the difference between a real-time EMI measurement and its baseline signal. Mathematically, RMSD is defined as:

$$RMSD\ index = \sqrt{\frac{\sum_{i=1}^{N}(G_r - G_{bl})^2}{\sum_{i=1}^{N}(G_{bl})^2}} \tag{1}$$

where G_r and G_{bl} is the conductance signal amplitude at frequency i from real-time and baseline measurements, respectively. Our previous research demonstrated that, among various quantitative indices used in EMI-based monitoring, RMSD exhibited the highest correlation with concrete strength development, making it the most effective choice for signal characterization in this context[2].

S4. Principal Component Analysis (PCA) for Database-Wide Signal–Strength Relationship

To explore the relationship between EMI signals and concrete strength across the entire database, Principal Component Analysis (PCA) was performed on the collected EMI spectra. PCA is a linear dimensionality reduction technique that transforms high-dimensional data into orthogonal principal components (PCs), ranked by the amount of variance each component captures. Each EMI signal vector, consisting of amplitudes across frequencies, was projected into a 2D space defined by the first two principal components

(PC_1 and PC_2). The percentage values associated with each axis (e.g., PC_1: 65.41%) indicate the proportion of total variance in the original data explained by that component.

We compared two PCA projections: one using only the real-time EMI signals, and the other incorporating both baseline and real-time signals as inputs. The projection using only real-time signals showed scattered and irregular distributions, making it difficult to discern strength-related patterns. In contrast, the inclusion of baseline signals significantly improved the compactness and structure of the projection, with data points showing clearer trends related to strength progression. This further supports the necessity of the baseline mechanism to reduce sensor-to-sensor variation and enhance model interpretability.

REFERENCE

1. Su, Y., Han, G., Kong, Z., Nantung, T. & Lu, N. Embeddable Piezoelectric Sensors for Strength Gain Monitoring of Cementitious Materials : The Influence of Coating Materials. *Engineered Science* 1–33 (2020) doi:Doi: https://dx.doi.org/10.30919/es8d1114.
2. Su, Y. F., Han, G., Amran, A., Nantung, T. & Lu, N. Instantaneous monitoring the early age properties of cementitious materials using PZT-based electromechanical impedance (EMI) technique. *Constr Build Mater* 225, 340–347 (2019).
